# Allochthonous resources are less important for faunal communities on highly productive, small tropical islands

**DOI:** 10.1002/ece3.8035

**Published:** 2021-08-26

**Authors:** Sebastian Steibl, Robert Sigl, Sanja Blaha, Sophia Drescher, Gerhard Gebauer, Elif Gürkal, Frederic Hüftlein, Anna Satzger, Michael Schwarzer, Dimitri Seidenath, Jana Welfenbach, Raphael S. Zinser, Christian Laforsch

**Affiliations:** ^1^ Department Animal Ecology I and BayCEER University of Bayreuth Bayreuth Germany; ^2^ BayCEER—Laboratory of Isotope Biogeochemistry University of Bayreuth Bayreuth Germany

**Keywords:** beach wrack, food web, marine subsidies, stable isotope analysis

## Abstract

Ecosystems are interconnected by energy fluxes that provide resources for the inhabiting organisms along the transition zone. Especially where in situ resources are scarce, ecosystems can become highly dependent on external resources. The dependency on external input becomes less pronounced in systems with elevated in situ production, where only consumer species close to the site of external input remain subsidized, whereas species distant to the input site rely on the in situ production of the ecosystem. It is largely unclear though if this pattern is consistent over different consumer species and trophic levels in one ecosystem, and whether consumer species that occur both proximate to and at a distance from the input site differ in their dependency on external resource inputs between sites. Using stable isotope analysis, we investigated the dependency on external marine input for common ground‐associated consumer taxa on small tropical islands with high in situ production. We show that marine input is only relevant for strict beach‐dwelling taxa, while the terrestrial vegetation is the main carbon source for inland‐dwelling taxa. Consumer species that occurred both close (beach) and distant (inland) to the site of marine input showed similar proportions of marine input in their diets. This supports earlier findings that the relevance of external resources becomes limited to species close to the input site in systems with sufficient in situ production. However, it also indicates that the relevance of external input is also species‐dependent, as consumers occurring close and distant to the input site depended equally strong or weak on marine input.

## Graphical abstract

This work investigated the relevance of marine versus terrestrial resources for the faunal community of small tropical islands. While the beach fauna shows high proportions of marine resources to their diets, the inland fauna is largely independent of these subsidies and primarily utilizes terrestrial primary production. However, species that occurred throughout the insular ecosystems had either consistently high or low contributions of marine material to their diet, suggesting that the relevance of marine subsidies is also consumer‐dependent.

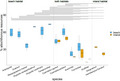

## 1 INTRODUCTION

Even when separated by distinct borders, ecosystems rarely function without interconnection to each other (Barrett et al., 2005). Cross‐ecosystem energy transfer is often crucial for animals and plants occurring in adjacent systems (Richardson & Wipfli, 2016). This external resource input into recipient ecosystems, commonly referred to as allochthonous input, can occur via biotic factors, when organisms forage and actively move between adjacent ecosystems (Bouchard & Bjorndal, 2000; Hilderbrand et al., 1999), or via abiotic factors, when wind, currents, or runoff passively transport resources from one ecosystem into another (Gauthier et al., 2011; Jansson et al., 2000; Richardson & Sato, 2015).

Numerous studies demonstrated the importance of this allochthonous input in different systems and taxa, for example, between forests and freshwater systems (Helfield & Naiman, 2001; Nakano & Murakami, 2001), the canopy and understory (Pringle & Fox‐Dobbs, 2008), benthic and pelagic zones (Renaud et al., 2015), or marine and coastal systems (Dugan et al., 2003). The allochthonous input thereby subsidizes the recipient system and can result in significant increases in the overall biomass production and species abundance (Gounand et al., 2018; Polis & Hurd, 1996; Subalusky & Post, 2019).

These subsidization effects are suggested to become more pronounced with an increasing ratio of allochthonous‐to‐autochthonous resources (Marczak et al., 2007). This is particularly well documented for different arid or desert insular ecosystems, which offer only few in situ basal resources, compared to the highly productive adjacent oceans. Here, the allochthonous input, either in the form of beach wrack or as guano, is considered to be crucial for the stability of the whole insular food web (Polis et al., 1997). In these systems, various taxa, such as spiders, mice, or scorpions, depend on allochthonous input or its consumers (Anderson & Polis, 1998; Gauthier et al., 2011; Polis & Hurd, 1996). In these low‐productive insular ecosystems, the allochthonous input benefitted all investigated organisms, as even those who primarily occurred inland return to the coast to forage on allochthonous marine input (Anderson & Polis, 1999; Stapp et al., 1999).

However, when two systems have only shallow productivity gradients and the ratio between allochthonous‐to‐autochthonous resources is low, the overall dependency on subsidies becomes limited to consumer species close to the site of allochthonous input (Marczak et al., 2007). It is largely unclear if this pattern is consistent among different consumer species and different trophic levels within one ecosystem. When the relevance of allochthonous input becomes limited to consumers close to the site of resource input (Muehlbauer et al., 2014; Paetzold et al., 2008), then consumer species with a broad distribution range that occur close to as well as at a distance to the site of allochthonous input might differ in their dependency on allochthonous input.

In this study, we evaluate the importance of allochthonous input for common ground‐associated consumer species on highly productive tropical islands. We sampled common terrestrial insular animal taxa, comprising different trophic guilds, that occurred either close to the site of allochthonous input, that is, the beach, or at distance, that is, the inland, or throughout the entire island, that is, in both habitats. The study was carried out on four islands in the Lhaviyani Atoll, Republic of Maldives. These islands receive high amounts of annual rainfall and are classified as tropical moist forests (Gillespie et al., 2012). Most allochthonous input on these islands is deposited passively in the form of washed‐up seagrass and marine carrion, while seabirds or other high‐dispersal foragers that usually are the main depositor of allochthonous nutrients on oceanic islands are virtually absent (Anderson & Polis, 1999). We hypothesized that, due to the investigated islands’ high autochthonous production, allochthonous marine input is only a relevant subsidy for ground‐associated consumer species close to the beach, while those taxa occurring inland mainly rely on autochthonous production. To evaluate the relevance of allochthonous marine inputs for consumers in systems with high in situ production, we used stable isotope mixing models based on carbon (δ^13^C) and nitrogen (δ^15^N) signatures to calculate the relative proportion of diet derived from allochthonous marine resources for each consumer taxon.

## 2 MATERIAL AND METHODS

### 2.1 Sampling location

Oceanic islands can be considered as discrete community assemblages, with oceans acting as barriers (Leibold et al., 2004; Mehranvar & Jackson, 2001). Multiple uninhabited islands of an atoll can thereby be treated as repetitive units (Steibl & Laforsch, 2019a). We investigated four small uninhabited islands in the Lhaviyani atoll (Republic of the Maldives), namely Dhidhdhoo, Gaaerifaru, Vavvaru, and Veyvah, (*N* = 4). We determined the islands’ sizes by walking along the shoreline of each island using GPS (Garmin eTrex Vista Cx; Garmin International Inc., Olathe, USA). The circumferences and areas of the four islands were 2,400 m and 116,537 m^2^ for Dhidhdhoo; 862 m and 29,081 m^2^ for Gaaerifaru; 855 m and 29,629 m^2^ for Vavvaru; and 706 m and 28,456 m^2^ for Veyvah. We conducted sampling between 26/05/2018 and 29/05/2018, sampling one island per day between 9:00 a.m. and 3:00 p.m. As the investigated islands lie in the tropics close to the equator with stable temperatures and wind conditions throughout the year, they show only little seasonal variation in primary production compared to more temperate systems (Clark et al., 2001), suggesting that variation in primary production should be minimal.

### 2.2 Sampling of allochthonous and autochthonous resources

To quantify the amount of standing stock at the shoreline and collect basal resources on the beach, we positioned five 10‐m transects randomly along each islands’ high tide drift line. In each 10 m‐transect, we placed four subplots (0.5 × 0.5 m) at the zero‐, three‐, six‐, and nine‐meter markings along the coastline and collected all organic material in the top 2 cm layer of the drift line. We categorized the collected material as either seagrass, marine carrion (i.e., washed‐up dead sea urchins and fish), or terrestrial debris. Undefined debris was excluded from further analysis as it accounted for only 0–0.2 g/m^2^ (Table 1). We weighed larger material on‐site using a scale (Etekcity EL11, Etekcity Corp.) and smaller material in the laboratory using a fine scale (TS‐300, G&G GmbH). We took five tissue samples from each resource category per island, transferred the samples to 1.5‐ml Eppendorf safe‐lock tubes (Eppendorf AG), and stored them at −20°C in a freezer until further processing.

**TABLE 1:** Allochthonous and autochthonous resource input on the beaches

Island	Seagrass [g/m^2^]	Marine carrion [g/m^2^]	Terrestrial material [g/m^2^]	Undefined debris [g/m^2^]	Total beach wrack [g/m^2^]
Dhidhdhoo	18.2 ± 8.6	0.0 ± 0	62.6 ± 8.7	0.1 ± 0.1	80.6 ± 15.8
Gaaerifaru	740.1 ± 213.7	0.0 ± 0	694.5 ± 625.6	0.0 ± 0.0	1,434.6 ± 569.4
Vavvaru	13.9 ± 8.4	0.0 ± 0	487.3 ± 215.1	0.1 ± 0.1	501.3 ± 211.6
Veyvah	4.4 ± 1.31	8.0 ± 7.6	556.7 ± 349.9	0.2 ± 0.2	569.3 ± 348.1

Notes

Amount of the different types of allochthonous input and accumulating terrestrial debris along the drift line for each of the four investigated islands (*N* = 4*;* mean ± standard error).

To sample terrestrial plant material, we collected five leaves from the dominant plant species that occurred on all four investigated islands (*Calophyllum inophyllum*, *Cassytha filiformis*, *Cyperus dubius*, *Launaea sarmentosa*, *Pandanus tectorius*, *Pemphis acidula*, *Scaevola taccada*, *Sesuvium portulacastrum*, *Suriana maritima*, *Tournefortia argentea*, *Wollastonia biflora*). We stored the leaves in paper bags and dried them at room temperature until further processing.

### 2.3 Consumer sampling

To investigate differences in marine subsidization between beach and inland consumer species on small tropical islands, we defined the beach habitat as ranging from the drift line up to the first 10 m of pioneer plant cover landwards, and the inland habitat as starting at a minimum 20 m away from beach and having shrub or tree vegetation. We obtained tissue samples from insects and spiders by collecting whole animals with insect nets (mesh size 1 mm). We obtained tissue samples from decapod crustaceans by grabbing the third walking leg of a crab with forceps and cutting it above the second tibia segment. This procedure has minimal impact on the crustaceans as the removed segments will be regenerated within the next molts (Kuris & Mager, 1975; Skinner, 1985; Skinner & Graham, 1970). We obtained tissue samples from the common house gecko, *Hemidactylus frenatus*, by grabbing its tail with forceps until caudal autotomy, that is, shedding of its tail, was initiated. Where possible, we collected five tissue samples per taxon in both habitats and on each island. If an investigated taxon was found in only one habitat, we collected five tissue samples only in the occupied habitat on each island. Because the Maldivian atolls are overall scarce in terrestrial taxa, especially vertebrates, compared to continental tropical ecosystems (Thaman, 2008), we could include all relevant and abundant macroinvertebrate and vertebrate groups that commonly occur on the investigated islands. We only excluded flying insect taxa, that is, moths, day‐active Lepidopterans, and Apidae, from the sampling as they are known pollinators that are obligatory herbivores and feed on plant pollen or nectar. We grouped the collected euarthropod taxa into the following taxonomic groups: Amphipoda (sandhoppers), Blattodea (cockroaches), Caelifera (locusts), Arachnida (spiders), Curculionidae (weevils), Formicidae (ants), Gryllidae (crickets), Spirobolida (millipedes), and Tenebrionidae (darkling beetles). We identified all decapods except *Grapsus* sp. (shore crab) to species level. We found and collected amphipods, Tenebrionidae, *Grapsus* sp., *Metopograpsus messor* (grapsid crab), *Ocypode ceratophthalmus*, and *O*. *cordimana* (both ghost crabs), if present on the investigated islands, only in the beach habitat. We found and collected Curculionidae, *Hemidactylus frenatus* (house gecko), and *Geograpsus grayii* (shore crab), if present on the investigated islands, only in the inland habitat. We found and collected Spirobolida, Caelifera, Blattodea, *Coenobita perlatus, C. rugosus* (two terrestrial hermit crab species), Formicidae, Gryllidae, and Arachnida, if present on the investigated islands, in the beach and inland habitat (Table 2). We sampled most species on all investigated islands, except for Gryllidae and *O*. *ceratophthalmus* (only Dhidhdhoo), Curculionidae (only Vavvaru), and Tenebrionidae (only Vavvaru and Dhidhdhoo). As all investigated taxa are ground‐associated, they cannot migrate between the islands. Therefore, their isotope signature can be considered as an integration of their diet obtained only from the investigated island, hence minimizing any mismatch between baseline and consumer data. We stored all collected consumer samples in 1.5‐ml Eppendorf safe‐lock tubes at −20°C until further processing.

**TABLE 2:** Isotope signature of the four basal resources

Island	Allochthonous resources		Autochthonous resources	
	Seagrass	Marine carrion	Terrestrial debris	Plant material
Dhidhdhoo	δ^13^C = −9.5 ± 3.0‰ δ^15^N = 2.9 ± 0.9‰	–	δ^13^C = −27.5 ± 1.6‰ δ^15^N = −1.7 ± 2.3‰	δ^13^C = −24.3 ± 6.8‰ δ^15^N = 1.0 ± 2.9‰
Gaaerifaru	δ^13^C = ‐6.7 ± 0.7‰ δ^15^N = 4.1 ± 1.9‰	–	δ^13^C = −28.8 ± 2.4‰ δ^15^N = 1.5 ± 1.2‰	δ^13^C = −24.7 ± 6.8‰ δ^15^N = 0.2 ± 3.0‰
Vavvaru	δ^13^C = −8.1 ± 1.8‰ δ^15^N = 3.0 ± 0.5‰	–	δ^13^C = −28.2 ± 2.0‰ δ^15^N = −2.8 ± 1.3‰	δ^13^C = −24.1 ± 6.8‰ δ^15^N = −1.0 ± 4.1‰
Veyvah	δ^13^C = −9.8 ± 1.3‰ δ^15^N = 2.0 ± 1.1‰	δ^13^C = −4.7 ± 2.8‰ δ^15^N = 3.8 ± 0.6‰	δ^13^C = −28.1 ± 0.7‰ δ^15^N = −3.0 ± 1.2‰	δ^13^C = −24.8 ± 6.4‰ δ^15^N = −0.9 ± 3.1 ‰
Average	δ^13^C = −8.6 ± 2.1‰ δ^15^N = 2.9 ± 1.2‰	–	δ^13^C = −28.1 ± 0.7‰ δ^15^N = −3.0 ± 1.2‰	δ^13^C = −24.5 ± 6.7‰ δ^15^N = −0.2 ± 3.3‰

Notes

For each island, the mean ± *SD* isotope signature is presented (*N* = 5 per island). As the isotope signatures did not differ significantly between the four investigated islands (PERMANOVA: *F* = 2.257, *df* = 3, *p* = .056), the average isotope signature was calculated over the four islands and used as baseline resource values in the Bayesian mixing models.

### 2.4 Stable isotope analysis

In the laboratory, we lyophilized all samples in a drying oven (Memmert GmbH + Co. KG, Schwabach, Germany) at 110°C for 48 hr and homogenized the dried samples using a ball mill (Retsch MM 400, Haan, Germany) at 30 Hz for 90 s. As recent findings suggest that acidification to remove inorganic carbon from samples of shoreline species for stable isotope analysis has no relevant effect on carbon isotope values, we analyzed the collected consumer tissue samples without acidification treatment (Pires‐Teixeira et al., 2020).

We measured the relative nitrogen and carbon isotope natural abundances of the tissue samples in a dual element analysis with an elemental analyzer (Carlo Erba Instruments 1108), coupled to a continuous flow isotope ratio mass spectrometer (delta S, Finnigan MAT, Bremen, Germany) via a ConFlo III open‐split interface (Thermo Fisher Scientific). Measured relative isotope abundances are denoted as δ values using (*R*_sample_/*R*_standard_−1) × 1,000 [‰], with *R*_sample_ and *R*_standard_ being the ratios of the heavy to light isotope of the sample and the standard, respectively. We calibrated standard gases with respect to international standards (CO_2_ vs. V‐PDB, N_2_ vs. N_2_ in the air) with the reference substances CH6, CO8, and NBS18 for carbon isotopes and N1 and N2 for nitrogen isotopes, provided by the International Atomic Energy Agency, Vienna, Austria (Bidartondo et al., 2004). We corrected the obtained δ^13^C values for their lipid content following the method described by Post et al. (2007) using linear regression equations to adjust the δ^13^C based on C:N ratios for terrestrial animals and relative carbon content for plant material with carbon content <40%, respectively. The corrected isotope signatures for each consumer are presented in Table S1.

### 2.5 Statistical analysis

We performed all statistical analysis using R 3.5.1 (R Core Team, 2018) extended with the “vegan” (Oksanen, 2015) and “simmr” packages (Parnell et al., 2010, 2013).

Prior to running the mixing models, we compared the isotope signature (δ^13^C and δ^15^N) of the four sampled basal resources (seagrass, marine carrion, terrestrial debris, terrestrial plant material) and the consumer species between the four investigated islands to test whether the four islands must be treated separately, or data can be combined using nonparametric PERMANOVA (4,999 permutations, Bray‐Curtis distance matrix). The isotope signatures of the basal resources did not differ significantly between the four investigated islands (*F* = 2.25, *df* = 3, *p* > .05) and were consequently averaged over the four investigated islands for the mixing model. The isotope signatures of the consumers differed significantly between species (*F* = 43.16, *df* = 16, *p* < .001) and between the four investigated islands (*F* = 10.64, *df* = 3, *p* < .001). Therefore, we treated the consumer species from each island separately in the mixing models.

For herbivorous and detritivorous consumers, we used trophic enrichment factors (TEFs) for terrestrial consumers without acidification treatment of +0.5 ± 0.17‰ for ^13^C and +2.4 ± 0.24‰ for ^15^N (McCutchan et al., 2005). For omnivorous and carnivorous consumers, we doubled the TEF and calculated the variability using (2 × *SD* ^2^)^0.5^ following the method described in Neres‐Lima et al. (2016) and Neves et al. (2021).

We run two separate Bayesian mixing models for herbivorous + detritivorous and for omnivorous + carnivorous consumers, respectively, using “simmr” version 0.4.5 to infer the relative contributions of the different allochthonous (seagrass, marine carrion) and autochthonous (terrestrial plant material, terrestrial detritus) resources for the consumer species in the beach and inland habitat on the four investigated islands. Prior to running the Bayesian mixing models, we visually inspected the data on whether the consumer species fall within the isotope polygon of the resources (Smith et al., 2013). Isotope signatures of Spirobolida, Curculionidae, Caelifera, and Blattodea fell outside the source polygon, and these consumers were thus excluded from the Bayesian mixing models. The Markov Chain Monte Carlo chains for both models were run with 1,000,000 iterations, discarding the first 500,000 runs. We tested the models’ convergences using Gelman‐Rubin diagnostics, and all MCMC runs showed acceptable model convergence (Gelman‐Rubin values between 1.00 and 1.02). As the aim of this study was to compare the relevance of allochthonous versus autochthonous resources for consumer species of tropical islands and the Bayesian mixing model indicted overall high correlation in the isotope signature between seagrass and marine carrion, and between plant material and terrestrial debris, we applied the posterior combining function on the two source pairs and grouped them in allochthonous and autochthonous resources, respectively. To test for differences in the relevance of allochthonous input for consumer species among habitats, we calculated the probabilities that the posterior probability of the relative contribution of allochthonous resources of each consumer was indeed greater than the relative contribution of autochthonous resources between beach and inland habitat. We assumed a significantly higher contribution of allochthonous resources in one habitat for probabilities >0.95. To further test for differences in contribution of allochthonous resources to the diets of the investigated consumers between species and between habitats, we extracted the estimated mean contributions of allochthonous resources for each consumer from the Bayesian mixing model. We compared the arcsin‐transformed relative contributions (Shapiro test for normality: *W* = 0.997, *p* = 0.380; Levene test for homoscedasticity: *F* = 0.943, *p* = 0.535) between species and habitat as explanatory variables using ANOVA with Tukey HSD post hoc testing and Bonferroni *p*‐value correction.

## 3 RESULTS

### 3.1 Quantification of allochthonous standing stock on the beach

The amount of allochthonous and autochthonous standing stock per m^2^ along the beach shoreline of the four investigated island ecosystems ranged between 18–740 g/m^2^ allochthonous material and 62–695 g/m^2^ autochthonous material (Table 1).

### 3.2 Differences in isotope signature between islands and consumer species

The isotope signature (δ^13^C and δ^15^N) of each of the four basal resources, that is, seagrass, marine carrion, terrestrial debris, and plant material, did not differ significantly between the four investigated islands (PERMANOVA: *F* = 2.257, *df* = 3, *p* = .056, Table 2). The isotope signatures (δ^13^C and δ^15^N) of the investigated consumers differed significantly between species (PERMANOVA: *F* = 43.160, *df* = 16, *p* < .001) and between the four investigated islands (*F* = 10.638, *df* = 3, *p* < .001), and the difference in isotope signature between species varied between the four investigated islands (interaction effect: *F* = 2.251, *df* = 33, *p* < .001).

### 3.3 Proportion of allochthonous resources in the diet of consumer species

**FIGURE 1:**
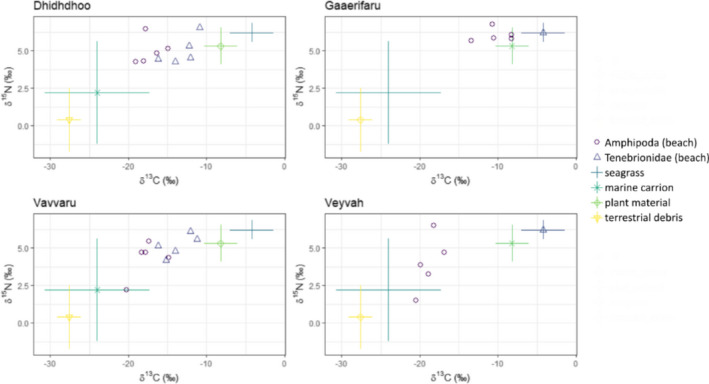
Isotope biplot for the primary consumers on the four investigated islands. The isotope signatures (δ^13^C and δ^15^N) are plotted for each primary consumer, that is, herbivores and detritivores, together with the mean isotope signature of the four basal resources from the four investigated islands (*N* = 5). Isospace plots have been corrected assuming 1 trophic enrichment factors (TEFs) for primary consumers, following the method described in Neres‐Lima et al. (2016) and Neves et al. (2021). Vertical and horizontal error bars indicate mean ± standard deviations of the four investigated basal resources

**FIGURE 2:**
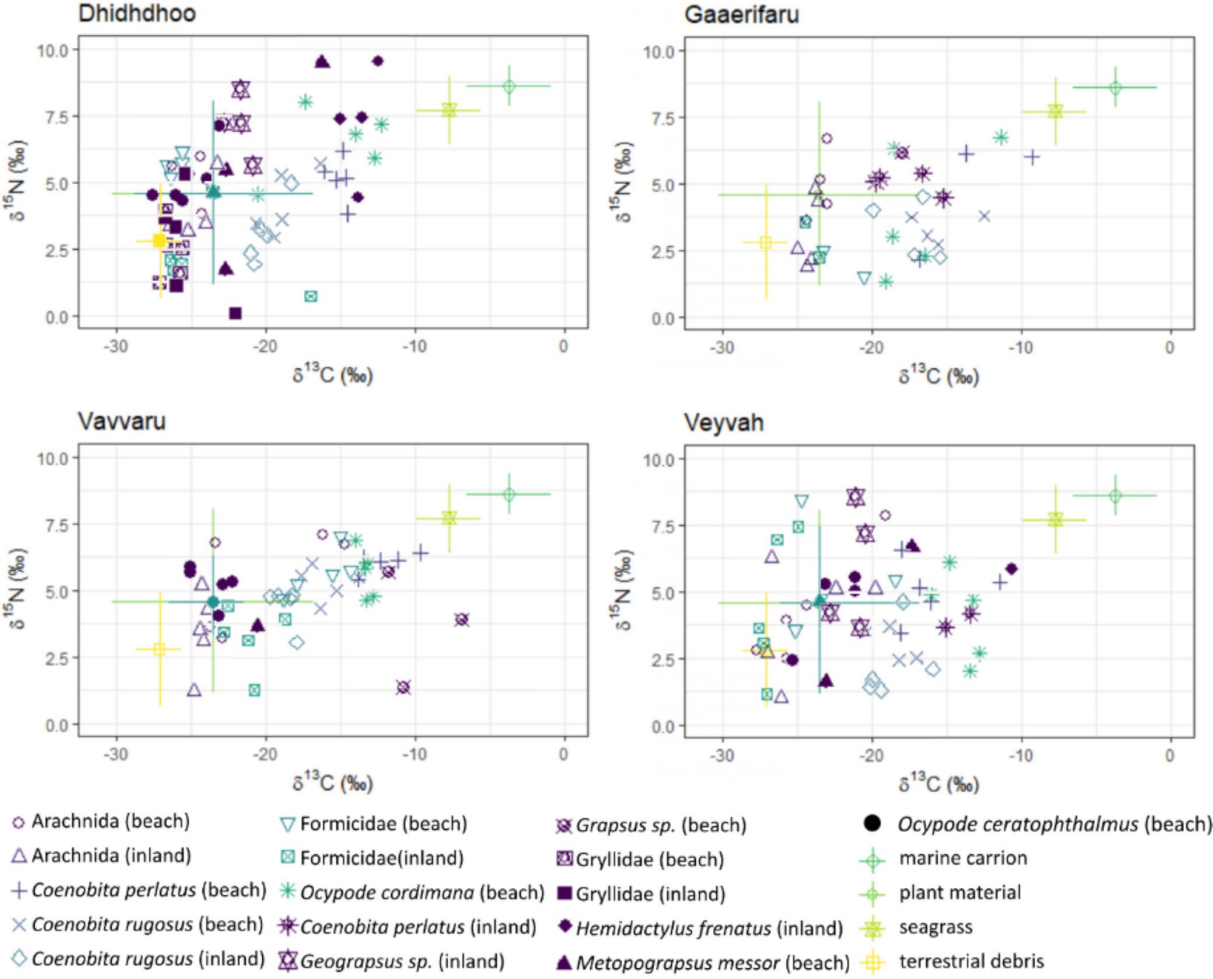
Isotope biplot for the secondary consumers on the four investigated islands. The isotope signatures (δ^13^C and δ^15^N) are plotted for each secondary consumer, that is, omnivores and carnivores, together with the mean isotope signature of the four basal resources from the four investigated islands (*N* = 5). Isospace plots have been corrected assuming 2 trophic enrichment factors (TEFs) for secondary consumers, following the method described in Neres‐Lima et al. (2016) and Neves et al. (2021). Vertical and horizontal error bars indicate mean ± standard deviations of the four investigated basal resources

The isotope signatures (δ^13^C and δ^15^N) of the different primary and secondary consumers on the four investigated islands ranged over the entire isotope source polygons formed by the four basal resources (Figures 1 and 2). The results from the Bayesian mixing models showed a great variability in the relative contribution of allochthonous resources (i.e., seagrass and marine carrion combined) among invertebrate consumer species but an overall consistency within species sampled in the beach and inland habitat, that is, either a high or a low proportion in both habitats (Table 3, Table S2). Only for Formicidae on two of the four investigated islands, the probability, that the estimated relative contribution of allochthonous resources to their diet is indeed greater in those specimens sampled at the beach than in those found inland, was >95%.

**TABLE 3:** Relative contributions of allochthonous resources to the diets of invertebrate consumers on the four investigated islands

Species	Habitat	Dhidhdhoo	Gaaerifaru	Vavvaru	Veyvah
Amphipoda	Beach	59.4 ± 5.6%	85.4 ± 4.3%	54.2 ± 7.1%	49.4 ± 7.2%
Tenebrionidae	Beach	74.8 ± 6.3%	–	72.6 ± 5.9%	–
Arachnida	Beach	18.5 ± 6.2%	23.4 ± 6.2%	34.4 ± 10.9%	18.3 ± 8.3%
	Inland	15.9 ± 6.3%	17.0 ± 5.6%	16.6 ± 5.3%	18.4 ± 8.6%
*Coenobita perlatus*	Beach	63.7 ± 8.4%	56.7 ± 19.4%	73.2 ± 10.0%	50.8 ± 12.9%
	Inland	–	44.1 ± 11.7%	–	54.4 ± 19.6%
*Coenobita rugosus*	Beach	41.9 ± 10.4%	36.5 ± 17.1%	56.3 ± 9.9%	44.4 ± 12.2%
	Inland	38.6 ± 9.0%	48.1 ± 13.9%	45.6 ± 8.9%	44.3 ± 10.5%
Formicidae	Beach	12.9 ± 5.2%	36.1 ± 17.3%	54.4 ± 9.2%	32.8 ± 14.2%
	Inland	17.2 ± 9.8%	29.8 ± 17.5%	30.6 ± 9.3% (*)	10.6 ± 5.9% (*)
*Geograpsus* sp.	Inland	32.4 ± 6.5%	–	35.3 ± 8.0%	–
*Grapsus* sp.	Beach	–	47.5 ± 11.4%	67.0 ± 22.3%	53.3 ± 19.4%
Gryllidae	Beach	7.6 ± 3.8%	–	–	–
	Inland	12.9 ± 6.2%	–	–	–
*H. frenatus*	Inland	17.1 ± 6.0%	–	23.1 ± 5.0%	24.9 ± 8.2%
*M. messor*	Beach	38.6 ± 15.0%	–	27.2 ± 10.1%	41.0 ± 18.3%
*O. ceratophthalmus*	Beach	70.5 ± 7.2%	–	–	68.4 ± 18.0%
*O. cordimana*	Beach	57.8 ± 10.5%	48.4 ± 13.9%	71.8 ± 7.4%	68.0 ± 9.1%

Notes

The contribution of allochthonous resources (a posteriori combination of seagrass and marine carrion) was estimated for each consumer species on each of the four investigated tropical islands individually using Bayesian mixing models. Values present mean ± standard deviation of relative contribution of allochthonous resources. Asterisks indicate >95% probability that the estimated relative contribution of allochthonous material to the diet of the consumer is indeed greater in the beach than the inland habitat (only for Formicidae on Vavvaru and Veyvah).

The relative contribution of allochthonous resources differed significantly between the different species (ANOVA: *F* = 15.581, *df* = 12, *p* < .001), but was not significantly different between habitats (*F* = 3.578, *df* = 1, *p* = .066), and the differences in relative contribution of allochthonous resources between the different species was not dependent on sampling location (interaction term species*habitat: *F* = 0.732, *df* = 0.575). Therefore, we must reject our initial hypothesis that consumer species on the beach have a consistently higher relative contribution of allochthonous resources in their diet than inland species. Instead, the contribution of allochthonous resources differed between consumer species, but not between the two insular habitats.

**FIGURE 3:**
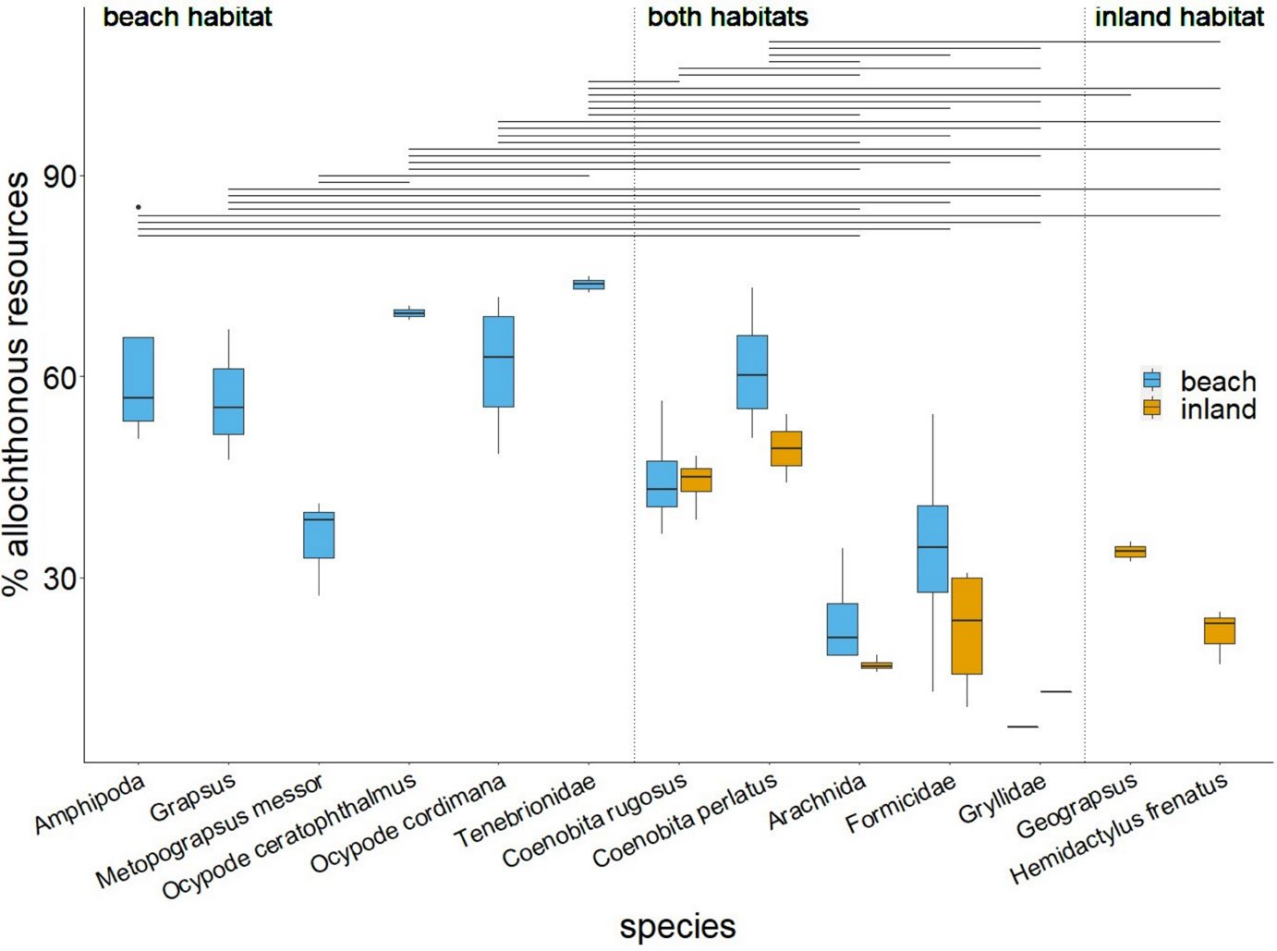
Contribution of allochthonous resources to the diet of insular invertebrates found in the beach, the inland, or in both habitats of tropical islands. The proportion of allochthonous resources, that is, seagrass and marine carrion, in the diet of common insular invertebrate consumer species occurring in the beach (blue), the inland (orange) or in both habitats was estimated based on δ^13^C and δ^15^N stable isotope ratios using Bayesian mixing models. For each consumer, the mean ± *SD* relative dietary contribution is presented based on the estimates from *N* = 4 investigated islands. Statistical differences (ANOVA, Tukey HSD post hoc test: *p* < .05) in the relative contribution between different consumer species are indicated by significance bars. The relative contribution of allochthonous resources did not differ significantly between habitats in species occurring in both habitats (*F* = 0.732, *df* = 4, *p* = .575)

On the one hand, consumer species that were found only in the beach habitat did show consistently higher relative contributions of allochthonous resources to their diet than consumer species sampled only in the inland (Tukey HSD: *p* < .05 for all pairwise comparisons; Figure 3). The mean contribution of allochthonous resources was >40% for all consumers that occurred exclusively on the beach, except *M*. *messor* (Table 3, Table S2). However, on the other hand, those consumer species that occurred in both habitats exhibited a less clear, bipartite pattern (Figure 3). The two hermit crab species, *C*. *rugosus* and *C*. *perlatus*, showed consistently greater relative contributions of allochthonous resources to their diets (>40%) in the inland and beach habitat than Arachnida, Formicidae, and Gryllidae from both habitats (Tukey HSD: *p* < .05 for all pairwise comparisons). Arachnida, Formicidae, and Gryllidae sampled at the beach and in the insular interior showed consistently low contributions (<25%) of allochthonous resources to their diet in both habitats.

## 4 DISCUSSION

Earlier research on the importance of allochthonous input has proposed an overall high dependency of insular food webs on marine input when donor and recipient systems differ markedly in their productivity (Fukami et al., 2006; Gauthier et al., 2011; Ince et al., 2007; Piovia‐Scott et al., 2011; Polis & Hurd, 1995; Spiller et al., 2010). The present study, conducted on islands with high terrestrial in situ production, partly supports the hypothesis that marine subsidies become only relevant for strict beach‐dwelling animal taxa that are close to the site of allochthonous input, while species occurring only inland primarily consume resources derived from the in situ primary production (Paetzold et al., 2008). However, we also show that consumer species with broader distribution ranges, that is, occurring both at the beach and inland, showed no difference in allochthonous subsidization between habitats. Instead, the proportion of allochthonous resources to the diets were either consistently high in both habitats (in the case of the terrestrial hermit crabs, *C*. *rugosus*, and *C*. *perlatus*), or consistently low (in the case of Formicidae, Gryllidae, and Arachnida), suggesting that relevance of allochthonous subsidization is also species‐dependent.

The primary carbon source for species on insular ecosystems can originate from the terrestrial vegetation (autochthonous resources) or the standing stock of deposited marine material along the beaches (allochthonous resources) (Barrett et al., 2005). In the present study, only strict beach‐dwelling taxa consumed high amounts of allochthonous material, while relying only to a smaller proportion on terrestrial‐derived resources. Although leaf litter was quantitatively more available than marine input in the standing stock of deposited material on the beaches, allochthonous resources strongly subsidized the strict beach‐dwelling taxa. As algal carbon is more easily digestible due to its lower structural stability than leaf litter, it is probably the preferred resource for beach‐dwelling detritivores (Marcarelli et al., 2011). The beach‐dwelling consumer species might additionally be restricted to beaches, because the environmental conditions or food availability further inland are unsuitable (Steibl et al., 2021). For example, low‐density sediment, required for burrowing, solidifies further landwards, therefore offering no opportunity to hide for strict beach dwellers (Burggren & McMahon, 1988; Rodrigues et al., 2016; da Rosa & Borzone, 2008; Steibl & Laforsch, 2019b).

On the other hand, the strictly inland‐dwelling taxa showed only small proportions of allochthonous resources in their diet. It suggests that these consumer species mainly rely on resources derived from the terrestrial in situ primary production and do not actively forage for allochthonous resources, probably because they cannot withstand the beach environment's physical conditions and therefore do not disperse into the beach habitat while foraging (McLachlan et al., 1993; Steibl & Laforsch, 2021).

*Coenobita rugosus* and *C*. *perlatus* were strongly subsidized by allochthonous resources and were found on the beach and the inland due to their ability and tendency to disperse landwards (Page & Willason, 1982). The consistently high contribution of allochthonous resources to their diet in both habitats suggests that terrestrial hermit crabs in the investigated insular system disperse landwards to seek shelter, but return to the shore to feed on allochthonous material (Hsu et al., 2018). The apparent movement of hermit crabs between the beach and inland might, however, be an important indirect subsidy for terrestrial plants or coprophagous inland consumers, when hermit crabs disperse landwards and release marine‐derived nutrients in the insular interior via defecation (Green et al., 1997; Schmitz et al., 2018).

Predatory spiders (Arachnida), omnivorous ants (Formicidae), and omnivorous crickets (Gryllidae) also commonly occurred on the beach and in the inland. However, these consumer species all had isotope signatures which suggest that they rely primarily on resources derived from autochthonous sources, even though they disperse further into the beach habitat (Colombini et al., 2011). Here, they might utilize the washed‐up detritus originating from terrestrial primary production, for example, coconuts. In this line, predatory spiders may also follow their prey, for example, ants, to the beach, which would then explain their “autochthonous” isotope signature and their occurrence in the beach habitat (Almquist, 1973). The estimated low contribution of allochthonous resources to the diet of spiders collected on the beaches suggests that they do not disperse to the beach to prey on the beach‐dwelling taxa subsidized by allochthonous resources.

These results are contrasting earlier findings from low‐productive insular ecosystems, in which all investigated insular consumers strongly depended on marine input (Polis & Hurd, 1996). Other than these desert islands, the Maldivian islands at focus in the present study are located in a tropical region with high annual precipitation (2,013 mm in the investigated atoll of this study vs. 59 mm on the investigated desert islands in the studies of Polis & Hurd, 1996), which strongly enhances in situ primary production (Gischler et al., 2014; Rosenzweig, 1968). The high autochthonous production of the tropical moist forests might be sufficient to allow most taxa to become independent of allochthonous input, while only the strict beach‐dwelling species remain dependent on allochthonous subsidies (Gillespie et al., 2012). Another noteworthy feature that might further result in the overall low dependency of the inland‐associated consumer species on allochthonous input is that no seabirds roosted or bred on the investigated islands. One of the most relevant links between allochthonous material and insular food webs is guano (Anderson & Polis, 1999; Croll et al., 2005; Fukami et al., 2006; Young et al., 2010). Where bird colonies occur on islands, they introduce large amounts of marine resources on islands and, other than the passive deposition of seagrass at the shoreline, can transport these nutrients far inland. As seabirds were virtually absent in the investigated system, or rested solely at the beach and never inland, the effects of marine subsidies were thus limited only to the direct input of seagrass and other wrack onto the beach habitat, making it primarily available to the beach‐dwelling consumer species.

Overall, we show that small tropical islands with high autochthonous production depend only partly on allochthonous subsidies. Taken together with previous findings of a strong dependency on allochthonous subsidies in low‐productive desert or tundra islands (Croll et al., 2005; Gauthier et al., 2011; Stapp & Polis, 2003), our study comprising multiple animal taxa supports the hypothesis that the relevance of allochthonous resources becomes limited to an edge effect when in situ production is sufficient (Paetzold et al., 2008). Where the donor and recipient ecosystems differ significantly in their productivity and the ratio of allochthonous‐to‐autochthonous resources is high, the recipient ecosystem becomes more dependent on allochthonous input (Marczak et al., 2007; Polis et al., 1997). Vice versa, this means that when the inland vegetation provides sufficient resources relative to the marine input, beaches can become sinks for allochthonous input from the adjacent oceans and are no longer links between ocean and inland. However, we also show that the dependency on allochthonous resources is to some extent also species‐dependent, as those consumer species that occurred throughout the whole island did not switch their diet toward allochthonous resources when closer to the site of allochthonous nutrient input. Besides the ratio of allochthonous‐to‐autochthonous material in a system, this suggests that the trophic niches of its consumer species further influence the dependency of a system on subsidization.

## CONFLICT OF INTEREST

The authors declare not conflicting interests.

## AUTHOR CONTRIBUTION

**Sebastian Steibl:** Conceptualization (equal); Data curation (equal); Formal analysis (equal); Funding acquisition (equal); Investigation (equal); Methodology (equal); Project administration (equal); Resources (equal); Software (equal); Supervision (equal); Validation (equal); Visualization (equal); Writing‐original draft (equal); Writing‐review & editing (equal). **Robert Sigl:** Conceptualization (equal); Investigation (equal); Methodology (equal); Project administration (equal); Supervision (equal); Writing‐original draft (equal); Writing‐review & editing (equal). **Sanja Blaha:** Investigation (equal); Methodology (equal); Writing‐original draft (equal). **Sophia Drescher:** Investigation (equal); Methodology (equal); Writing‐original draft (equal). **Gerhard Gebauer:** Conceptualization (equal); Formal analysis (equal); Methodology (equal); Project administration (equal); Resources (equal); Validation (equal); Writing‐original draft (equal); Writing‐review & editing (equal). **Elif Gürkal:** Investigation (equal); Methodology (equal); Writing‐original draft (equal). **Frederic Hüftlein:** Investigation (equal); Methodology (equal); Writing‐original draft (equal). **Anna Satzger:** Investigation (equal); Methodology (equal); Writing‐original draft (equal). **Michael Schwarzer:** Investigation (equal); Methodology (equal); Writing‐original draft (equal). **Dimitri Seidenath:** Investigation (equal); Methodology (equal); Writing‐original draft (equal). **Jana Welfenbach:** Investigation (equal); Methodology (equal); Writing‐original draft (equal). **Raphael S. Zinser:** Investigation (equal); Methodology (equal); Writing‐original draft (equal). **Christian Laforsch:** Conceptualization (equal); Funding acquisition (equal); Investigation (equal); Methodology (equal); Project administration (equal); Resources (equal); Supervision (equal); Validation (equal); Writing‐original draft (equal); Writing‐review & editing (equal).

## Supporting information

Table S1‐S2

## Data Availability

All raw data and statistical codes can be accessed under Dryad Data repository https://doi.org/10.5061/dryad.5x69p8d1k.
