# Supplementary material for: Allochthonous resources are less important for faunal communities on highly productive, small tropical islands
Source: Ecol Evol. 2021 Aug 26;11(19):13128–38. doi: 10.1002/ece3.8035 (PMC8495779; doi:10.1002/ece3.8035)
Supplement: Supplementary file 1 — Table S1‐S2 [file ECE3-11-13128-s001.docx]

**Supplementary table S1: Stable isotope raw data of the investigated insular consumer species.** For each consumer species at each of the two sampling locations (beach, inland) and each of the four investigated islands, the mean ± SE δ^13^C and δ^15^N values are presented (N = 5 per consumer species).

|  | **Dhidhdhoo** | | **Gaaerifaru** | | **Vavvaru** | | **Veyvah** | |
| --- | --- | --- | --- | --- | --- | --- | --- | --- |
| **Species** | **δ^13^C** | **δ^15^N** | **δ^13^C** | **δ^15^N** | **δ^13^C** | **δ^15^N** | **δ^13^C** | **δ^15^N** |
| Amphipoda (beach) | -15.6 ± 0.7 | 5.0 ± 0.4 | -8.9 ± 0.8 | 6.0 ± 0.2 | -16.2 ± 0.7 | 4.3 ± 0.5 | -14.8 ± 0.9 | 4.0 ± 0.8 |
| Arachnida (beach) | -24.2 ± 0.5 | 5.2 ± 0.5 | -22.6 ± 0.5 | 4.9 ± 0.7 | -19.4 ± 1.8 | 5.5 ± 0.9 | -23.6 ± 1.3 | 4.3 ± 1.0 |
| Arachnida (inland) | -24.1 ± 0.8 | 3.9 ± 0.6 | -23.5 ± 0.2 | 3.1 ± 0.6 | -23.5 ± 0.3 | 3.5 ± 0.7 | -24.0 ± 1.4 | 4.0 ± 1.0 |
| Blattodea (beach) | -23.8 ± 2.0 | -0.2 ± 0.1 | -23.5 ± 0.9 | 1.6 ± 1.1 | -22.8 ± 0.4 | -2.0 ± 0.6 | -26.5 ± 0.5 | 0.0 ± 0.8 |
| Blattodea (inland) | - | - | -24.4 ± 0.2 | -0.3 ± 0.8 | -22.8 ± 0.5 | 1.1 ± 1.5 | -24.6 ± 0.6 | 1.0 ± 0.7 |
| Caelifera (beach) | -24.7 ± 1.5 | 3.6 ± 0.2 | -16.6 ± 2.5 | 1.7 ± 0.5 | -22.6 ± 0.5 | 1.5 ± 1.0 | -19.4 ± 2.5 | 1.4 ± 0.8 |
| Caelifera (inland) | -19.3 ± 4.0 | -0.2 ± 0.5 | - | - | - | - | -12.6 ± 0.9 | 0.6 ± 0.6 |
| *Coenobita perlatus* (beach) | -6.5 ± 1.9 | 5.1 ± 0.4 | -7.6 ± 2.8 | 4.8 ± 1.3 | -6.8 ± 0.8 | 6.1 ± 0.2 | -12.8 ± 1.4 | 5.0 ± 0.5 |
| *Coenobita perlatus* (inland) | - | - | -11.2 ± 0.9 | 5.0 ± 0.2 | - | - | -10.1 ± 0.8 | 3.9 ± 0.3 |
| *Coenobita rugosus* (beach) | -14.4 ± 0.9 | 4.2 ± 0.5 | -12.5 ± 3.0 | 2.6 ± 0.7 | -10.5 ± 0.6 | 5.3 ± 0.3 | -13.6 ± 0.9 | 3.0 ± 0.3 |
| *Coenobita rugosus* (inland) | -14.7 ± 0.3 | 3.1 ± 0.5 | -12.2 ± 1.4 | 3.3 ± 0.6 | -13.3 ± 1.1 | 4.4 ± 0.3 | -14.0 ± 0.9 | 2.2 ± 0.6 |
| Curculionidea (beach) | - | - | - | - | -23.2 ± 0.2 | -1.5 ± 0.7 | - | - |
| Formicidae (beach) | -22.6 ± 0.6 | 4.4 ± 1.3 | -20.3 ± 1.3 | 2.0 ± 0.5 | -14.3 ± 0.7 | 5.7 ± 0.4 | -21.0 ± 2.1 | 5.8 ± 1.4 |
| Formicidae (inland) | -22.2 ± 1.6 | 1.8 ± 0.3 | -20.4 ± 0.2 | 2.9 ± 0.7 | -18.5 ± 1.0 | 3.2 ± 0.5 | -24.0 ± 0.6 | 4.4 ± 1.2 |
| *Geograpsus* (inland) | -20.7 ± 0.4 | 6.6 ± 0.7 | - | - | - | - | 19.0 ± 0.8 | 5.9 ± 1.2 |
| *Grapsus* (beach) | - | - | - | - | -8.0 ± 1.4 | 3.6 ± 1.3 | -12.7 ± 0.9 | 5.2 ± 0.6 |
| Gryllidae (beach) | -25.5 ± 0.4 | 2.4 ± 0.5 | - | - | - | - | - | - |
| Gryllidae (inland) | -24.4 ± 1.1 | 2.7 ± 0.9 | - | - | - | - | - | - |
| *Hemidactylus frenatus* (inland) | -22.9 ± 0.7 | 5.1 ± 0.5 | - | - | -20.0 ± 1.1 | 5.2 ± 0.3 | -22.4 ± 1.0 | 4.6 ± 0.7 |
| *Metopograpsus messor* (beach) | -18.8 ± 2.1 | 5.5 ± 2.2 | - | - | - | - | -17.6 ± 4.1 | 4.1 ± 2.5 |
| *Ocypode ceratophthalmus* (beach) | -11.2 ± 0.3 | 7.2 ± 1.1 | - | - | - | - |  |  |
| *Ocypode cordimana* (beach) | -14.1 ± 1.5 | 6.5 ± 0.6 | -14.7 ± 1.6 | 3.9 ± 1.1 | -12.0 ± 0.3 | 5.6 ± 0.4 | -11.4 ± 1.0 | 4.1 ± 0.8 |
| Spirobolida (beach) | -19.9 ± 0.3 | 0.7 ± 1.3 | -17.0 ± 0.5 | -1.8 ± 0.5 | -19.1 ± 0.2 | -2.8 ± 0.4 | -22.4 ± 0.2 | -2.4 ± 0.4 |
| Spirobolida (inland) | -20.1 ± 0.2 | -1.8 ± 0.2 | -16.6 ± 0.6 | -2.0 ± 0.5 | -19.4 ± 0.2 | -2.6 ± 0.4 | -20.6 ± 0.4 | -3.3 ± 0.5 |
| Tenebrionidae (beach) | -11.7 ± 0.9 | 5.0 ± 0.4 | - | - | -12.6 ± 0.9 | 5.1 ± 0.3 | - | - |

**Supplementary table S2: Quantiles of the estimated relative contributions of allochthonous resources.** For each species and for each of the four investigated islands, the 2.5%, 50% (i.e., the mode), and 97.5% of the estimated relative contributions of allochthonous resources to the consumers’ diet based on Bayesian stable isotope mixing model (δ^13^C and δ^15^N) is presented. Consumer species that fell outside the resource polygon were removed from the model and are thus not included in this table (see methods section).

|  | **Dhidhdhoo** | | | **Gaaerifaru** | | | **Vavvaru** | | | | **Veyvah** | | |
| --- | --- | --- | --- | --- | --- | --- | --- | --- | --- | --- | --- | --- | --- |
| **Species** | **2.5%** | **50%** | **97.5%** | **2.5%** | **50%** | **97.5%** | **2.5%** | **50%** | **97.5%** | **2.5%** | | **50%** | **97.5%** |
| Amphipoda (beach) | 46.8% | 59.7% | 69.6% | 76.2% | 85.5% | 92.9% | 38.5% | 54.9% | 66.8% | 32.9% | | 50.2% | 61.5% |
| Tenebrionidae (beach) | 60.7% | 75.6% | 85.6% | - | - | - | 58.8% | 73.1% | 82.5% | - | | - | - |
| Arachnida (beach) | 8.2% | 18.0% | 32.0% | 11.5% | 23.3% | 36.4% | 13.9% | 34.6% | 56.4% | 5.5% | | 17.2% | 37.3% |
| Arachnida (inland) | 6.1% | 15.3% | 30.0% | 6.5% | 17.0% | 27.5% | 6.9% | 16.6% | 27.4% | 5.7% | | 17.0% | 38.9% |
| *Coenobita perlatus* (beach) | 39.1% | 65.7% | 74.1% | 15.0% | 61.4% | 86.5% | 44.7% | 75.7% | 85.8% | 21.4% | | 53.2% | 70.4% |
| *Coenobita perlatus* (inland) | - | - | - | 18.5% | 45.5% | 63.4% | - | - | - | 12.7% | | 58.8% | 84.7% |
| *Coenobita rugosus* (beach) | 17.0% | 43.9% | 57.0% | 6.5% | 36.5% | 70.0% | 29.6% | 58.4% | 69.7% | 13.5% | | 47.1% | 62.6% |
| *Coenobita rugosus* (inland) | 15.0% | 40.7% | 51.3% | 14.0% | 51.2% | 68.6% | 21.4% | 47.9% | 57.5% | 16.3% | | 46.6% | 58.9% |
| Formicidae (beach) | 4.5% | 12.4% | 24.7% | 8.8% | 34.2% | 76.6% | 33.0% | 55.9% | 68.6% | 10.3% | | 31.0% | 67.1% |
| Formicidae (inland) | 3.5% | 15.7% | 41.2% | 7.1% | 25.5% | 74.9% | 10.8% | 31.9% | 46.1% | 3.1% | | 9.5% | 25.1% |
| *Geograpsus* (inland) | 19.6% | 32.3% | 45.2% | - | - | - | - | - | - | 18.0% | | 35.8% | 49.9% |
| *Grapsus* (beach) | - | - | - | 19.9% | 48.8% | 65.8% | 14.3% | 75.4% | 92.6% | 14.8% | | 59.4% | 85.0% |
| Gryllidae (beach) | 2.4% | 7.0% | 16.2% | - | - | - | - | - | - | - | | - | - |
| Gryllidae (inland) | 4.0% | 12.1% | 26.9% | - | - | - | - | - | - | - | | - | - |
| *Hemidactylus frenatus* (inland) | 7.0% | 16.5% | 30.7% | - | - | - | 13.7% | 23.0% | 33.0% | 10.5% | | 24.6% | 42.4% |
| *Metopograpsus messor* (beach) | 11.3.% | 37.9% | 72.1% | - | - | - | 8.2% | 26.9% | 47.1% | 9.9% | | 39.3% | 81.7% |
| *Ocypode ceratophthalmus* (beach) | 52.5% | 71.6% | 81.7% | - | - | - | - | - | - | 25.2% | | 73.6% | 92.1% |
| *Ocypode cordimana* (beach) | 35.8% | 58.6% | 76.8% | 15.6% | 50.7% | 70.5% | 50.1% | 73.3% | 81.3% | 40.5% | | 70.1% | 79.1% |
